# Antidepressant effects and therapeutic potential of naringenin: a systematic review and meta-analysis of preclinical studies

**DOI:** 10.3389/fphar.2026.1836030

**Published:** 2026-06-24

**Authors:** Miaomiao Huang, Dan Xiao, Jiali Wu, Mengsi Chen, Pan Du, Yi Yuan

**Affiliations:** 1 Department of Integrated Traditional Chinese and Western Medicine, The Second People’s Hospital of Yibin, Yibin City, China; 2 Department of Traditional Chinese Medicine Rehabilitation, The People’s Hospital of Zitong County, Mianyang City, China

**Keywords:** depression, inflammatory response, naringenin, neurotransmitters, oxidative stress

## Abstract

**Background:**

The prevalence of depression has increased steadily over recent decades, making it a major global public health concern. Although currently available pharmacotherapies demonstrate therapeutic efficacy, their clinical utility is often limited by adverse effects. Naringenin, a naturally occurring flavonoid compound, has shown potential antidepressant-like effects in preclinical studies. However, no comprehensive meta-analysis has yet systematically evaluated its efficacy in animal models of depression.

**Objective:**

This study aimed to systematically assess the antidepressant-like effects of naringenin in animal models of depression through a systematic review and meta-analysis, and to further elucidate its underlying mechanisms of action.

**Methods:**

A systematic literature search was conducted across five electronic databases (Embase, Web of Science, PubMed, Cochrane Library, and OVID) from database inception to November 2025. Methodological quality was assessed using the SYRCLE risk of bias tool. Data synthesis and statistical analyses were performed using Review Manager 5.4.

**Results:**

A total of 13 studies involving 212 rats were included. Meta-analysis demonstrated that, compared with the control group, naringenin treatment significantly reduced immobility time in the forced swimming test (FST)[SMD = −4.66; 95% CI (−6.55, −2.76); P < 0.00001] and tail suspension test (TST) [SMD = −5.36; 95% CI (−7.37, −3.36); P < 0.0001], increased the number of crossings in the open field test (OFT) [SMD = 2.18; 95% CI (0.52, 3.85); P= 0.01], and elevated sucrose consumption [SMD = 4.18; 95% CI (2.66, 5.71); P <0.00001]. Furthermore, naringenin reduced levels of tumor necrosis factor-alpha (TNF-α) and interleukin-1 beta (IL-1β), restored norepinephrine (NE) and serotonin (5-HT) concentrations, increased superoxide dismutase (SOD), catalase (CAT), and glutathione (GSH) levels, decreased malondialdehyde (MDA) and nitrite (NIT) levels, upregulated brain-derived neurotrophic factor (BDNF) expression, and lowered serum corticosterone(CORT) levels. Although heterogeneity was observed in behavioral outcomes, sensitivity analyses confirmed the robustness of the pooled results.

**Conclusion:**

Naringenin ameliorates depressive-like behaviors in rats, potentially through anti-inflammatory effects, modulation of neurotransmitter levels, and attenuation of oxidative stress. Nevertheless, all current evidence is derived from animal experiments. High-quality human clinical trials are needed in the future to evaluate its therapeutic efficacy.

## 1 Introduction

Depression is a prevalent psychiatric disorder characterized primarily by persistent low mood, diminished interest or pleasure, psychomotor retardation, cognitive impairment, and somatic symptoms; in severe cases, it may lead to suicidal ideation or behavior. The World Health Organization (WHO) estimates that 5.7% of adults worldwide live with depression (Organnization, 2025). According to the Global Burden of Disease Study 2021, approximately 33.241 million people were living with depression, which accounted for the highest proportion of disability-adjusted life years among mental disorders (36.24%) (Rong et al., 2025). Projections based on the Bayesian age-period-cohort model indicate that by 2040, the global age-standardized incidence rate of depression will rise to 8,478.2 per 100,000 population (Xu et al., 2025). Collectively, these findings underscore that depression has become a major global public health challenge. Psychological intervention and drug therapy are the mainstays of current depression management. Traditional antidepressants are associated with prominent adverse effects, including emotional numbness (71%), sexual dysfunction (66%), suicidal ideation (50%) and drug withdrawal symptoms (59%) (John and James, 2018). For this reason, natural neuroprotective agents featuring safety, low toxicity and multi-target activity have emerged as an important research avenue for new antidepressant drugs. All abbreviations used in this paper are listed in Table 1.

**TABLE 1:** List of acronyms.

Abbreviation	Full term
BDNF	Brain-derived neurotrophic factor
CORT	Corticosterone
CIs	Confidence intervals
FST	Forced swimming test
GSH	Glutathione
IL-1β	Interleukin-1 beta
IFN-γ	Interferon-γ
LPS	Lipopolysaccharide
MDA	Malondialdehyde
MAPK14	Mitogen-activated protein kinase 14
NE	Norepinephrine
NF-κB	Nuclear factor-κB
OFT	Open field test
ROS	Reactive oxygen species
SPT	Sucrose preference test
SMDs	Standardized mean differences
TNF-α	Tumor necrosis factor-alpha
5-HT	Serotonin

Naringenin (4′,5,7-trihydroxyflavanone), a polyphenolic phytochemical of the flavanone subclass, occurs naturally in citrus fruits, along with bergamot, tomatoes, cocoa and cherries (Bhia et al., 2021). Naringenin exhibits multiple pharmacological activities, including anti-inflammatory, antioxidant, immunomodulatory activities, as well as improvements in mitochondrial function and regulation of the hypothalamic-pituitary-adrenal (HPA) axis (Amin et al., 2020; Zhi et al., 2025). Chronic stress overactivates the HPA axis, triggering excessive cortisol release and microglial hyperactivation; This process further initiates the nuclear factor-κB/mitogen-activated protein kinase 14 (NF-κB/MAPK14) inflammatory signaling pathway, facilitates the assembly and activation of the nucleotide-binding oligomerization domain-like receptor protein 3 (NLRP3) inflammasome, and induces the release of pro-inflammatory factors including tumor necrosis factor-α (TNF-α) and interleukin-1β (IL-1β); Meanwhile,the secretion of anti-inflammatory mediators such as neuropeptide Y (NPY) and interleukin-10 (IL-10) is suppressed (Hassamal, 2023; Woźny-Rasała and Ogłodek, 2025). Chronic inflammation further induces mitochondrial dysfunction, excessive reactive oxygen species (ROS) production and redox imbalance, thereby triggering neuronal oxidative damage, DNA strand breaks and cell apoptosis (Qin et al., 2024). Inflammation and oxidative stress act synergistically to suppress monoamine synthases and increase monoamine oxidase A (MAO-A) activity, thereby disrupting the balance of neurotransmitters including serotonin (5-HT), norepinephrine (NE) and dopamine (DA) (Liang et al., 2024; Saggu et al., 2025). Meanwhile, they downregulate the cAMP response element-binding protein/brain-derived neurotrophic factor (CREB/BDNF) signaling pathway, inhibit neurogenesis and synaptic plasticity, and ultimately induce depression-like behaviors (Dou et al., 2022; Liu et al., 2025). Preclinical studies, including *in vitro* cell experiments and animal models, indicate that the antidepressant potential of naringenin may be associated with the following mechanisms.

It inhibits the NF-κB/MAPK14 pathway (Batool et al., 2025; Solanki et al., 2025) and NLRP3 inflammasome activation, and reduces the production of pro-inflammatory cytokines (Abdelkawy et al., 2024; Bansal et al., 2018; Olugbemide et al., 2021; Umukoro et al., 2018), while upregulating anti-inflammatory factors (Abdelkawy et al., 2024; Zhang et al., 2023). Naringenin also restores the activities of antioxidant enzymes such as glutathione (GSH), superoxide dismutase (SOD) and catalase (CAT) in the hippocampus and cerebral cortex, thereby alleviating oxidative damage (Bansal et al., 2018; Olugbemide et al., 2021). Additionally, it suppresses MAO-A activity and elevates the levels of 5-HT, NE and DA (Hsu et al., 2025), and activates the BDNF-tropomyosin receptor kinase B (TrkB) pathway to exert neuroprotective effects (Zhang et al., 2023). It should be objectively noted that the commonly used tail suspension test (TST) and forced swim test (FST) only assess behavioral despair and stress coping capacity. These assays fail to fully recapitulate the complex pathology and multidimensional symptoms of major depressive disorder in humans, which greatly limits their clinical translation. Population-based cross-sectional studies have suggested that adequate dietary intake of flavonoids may be associated with a reduced risk of depression (Deng et al., 2024; Yang et al., 2024). However, such studies are prone to confounding factors and cannot establish causality, so their findings should be interpreted with caution. Despite substantial preclinical evidence supporting the antidepressant effects of naringenin, its clinical application is markedly hindered by unfavorable pharmacokinetic properties. Its poor water solubility leads to low overall bioavailability (Alam et al., 2014). Additionally, extensive intestinal first-pass metabolism (Orrego-Lagarón et al., 2015), low blood-brain barrier permeability (Stasiłowicz-Krzemień et al., 2022) and rapid *in vivo* clearance further impair its delivery efficiency to the central nervous system. While formulation strategies such as nanocarriers, liposomes and prodrugs have been investigated, no mature approaches have been established to date.

Current preclinical studies on naringenin for depression vary greatly in animal models, behavioral outcomes, dosages, treatment durations and detection indicators. The lack of quantitative synthesis and systematic evaluation makes it difficult to objectively assess its antidepressant effect size, time-effect relationship and dose-effect relationship. To date, no relevant meta-analysis has quantitatively synthesized the available preclinical data, resulting in unclear evidence strength and limited generalizability of existing findings. Accordingly, this study aims to conduct a meta-analysis to systematically integrate preclinical evidence. We hypothesize that naringenin ameliorates depression-like behaviors in rodents via synergistic multi-target mechanisms, including anti-inflammation, antioxidation, restoration of monoamine neurotransmitter homeostasis, activation of BDNF signaling, as well as promotion of neurogenesis and synaptic plasticity. This work will provide evidence-based references for subsequent clinical trials and pharmaceutical development.

## 2 Materials and methods

### 2.1 Search strategy

From database inception to November 2025, a comprehensive literature search was conducted across five electronic databases: Embase, Web of Science, PubMed, the Cochrane Library, and OVID. The search strategy was developed in accordance with the PICOS framework, with “depression” and “naringenin” defined as the core concepts. A combination of controlled vocabulary terms and free-text keywords was applied. Synonymous terms within each domain were combined using the Boolean operator OR, while different domains were linked using AND to refine the search scope. The detailed search strategy is presented in Tables 2-6. All searches were performed independently by two investigators. Language restrictions were not applied, and grey literature was not retrieved. This review was not registered with PROSPERO or any other database.

**TABLE 2:** Search strategy on PubMed.

#	Search	Result
1	(((((((((((((depression [MeSH terms]) OR (depressive Disorder [MeSH terms])) OR (depression [Title/Abstract])) OR (depressive Disorder [Title/Abstract])) OR (bipolar Disorder [Title/Abstract])) OR (depressive Symptom [Title/Abstract])) OR (emotional Depression [Title/Abstract])) OR (depressive Neuroses [Title/Abstract])) OR (depressive Neurosis [Title/Abstract])) OR (endogenous Depression [Title/Abstract])) OR (Melancholia [Title/Abstract])) OR (unipolar Depression [Title/Abstract])) OR (depressive Syndrome [Title/Abstract])) OR (neurotic Depression [Title/Abstract])	605,468
2	(((((naringenin [MeSH terms]) OR (naringenin [Title/Abstract])) OR (4′,5,7-trihydroxyflavanone [Title/Abstract])) OR (BE 14348 A [Title/Abstract])) OR (naringenin-7-sulfate [title/Abstract])) OR ((2 S)-naringenin [Title/Abstract]) “naringenin” [Title/Abstract] OR ″4 5 7 trihydroxyflavanone” [Title/Abstract] OR “be 14348a” [Title/Abstract] OR “naringenin-7-sulfate” [title/Abstract] OR (“2 S” [All fields] AND “naringenin” [Title/Abstract])	4,961
3	#1 AND #2	45

**TABLE 3:** Search strategy on Embase.

#	Search	Result
1	‘depression'/exp OR depression OR ‘depressive disorder'/exp OR ‘depressive disorder’ OR (depressive AND (‘disorder'/exp OR disorder)) OR ‘bipolar disorder'/exp OR ‘bipolar disorder’ OR (bipolar AND (‘disorder '/exp OR disorder)) OR ‘depressive symptom'/exp OR ‘depressive symptom’ OR (depressive AND (‘symptom'/exp OR symptom)) OR emotional depression OR ‘depressive neuroses’ OR (depressive AND (‘neuroses'/exp OR neuroses)) OR ‘depressive neurosis'/exp OR ‘depressive neurosis’ OR (depressive AND (‘neurosis'/exp OR neurosis)) OR endogenous depression OR ‘melancholia'/exp OR melancholia OR unipolar depression OR ‘depressive syndrome'/exp OR ‘depressive syndrome’ OR (depressive AND (‘syndrome'/exp OR syndrome)) OR neurotic depression	1,150,364
2	‘naringenin'/exp OR naringenin OR ‘4,5,7 trihydroxyflavanone':ti,ab,kw OR ‘be 14348a':ti,ab,kw OR ‘naringenin 7 sulfate':ti,ab,kw OR (2s:ti,ab,kw AND -naringenin:ti,ab,kw)	10,251
3	#1 AND #2	163

**TABLE 4:** Search strategy on Cochrane Library.

#	Search	Result
1	(depression):ti,ab,kw OR (depressive Disorder):ti,ab,kw OR (bipolar Disorder):ti,ab,kw OR (depressive Symptom):ti,ab,kw OR (emotional Depression):ti,ab,kw	133,137
2	(Depressive Neuroses):ti,ab,kw OR (depressive Neurosis):ti,ab,kw OR (endogenous Depression):ti,ab,kw OR (Melancholia):ti,ab,kw OR (unipolar Depression):ti,ab,kw	4,105
3	(Depressive Syndrome):ti,ab,kw OR (neurotic Depression):ti,ab,kw	12,086
4	#1 OR #2 OR #3	133,166
5	(naringenin):ti,ab,kw OR (BE 14348 A):ti,ab,kw	63
6	#4 AND #5	0

**TABLE 5:** Search strategy on Web of Science.

#	Search	Result
1	Depression (all fields) or depressive disorder (all fields) or bipolar disorder (all fields) or depressive symptom (all fields) or emotional depression (all fields) or depressive neuroses (all fields) or depressive neurosis (all fields) or endogenous depression (all fields) or melancholia (all fields) or unipolar depression (all fields) or depressive syndrome (all fields) or neurotic depression (all fields)	862,037
2	Naringenin (all fields) or 4′,5,7-trihydroxyflavanone (all fields) or BE 14348 A (all fields) or naringenin-7-sulfate (all fields) or (2 S)-naringenin (all fields)	7,197
3	#1 AND #2	64

**TABLE 6:** Search strategy on OVID.

#	Search	Result
1	(Depression or depressive disorder or bipolar Disorder).af	558,179
2	(Naringenin or 4′,5,7-trihydroxyflavanone or BE 14348 A or naringenin-7-sulfate).af	3,982
3	#1 AND #2	34

### 2.2 Study selection

Studies meeting the PICOS criteria were included in the analysis as follows: (1) Population (P): rodent models of depression (Including: dexamethasone-induced, cadmium-induced, corticosterone-induced, finasteride-induced, bilateral olfactory bulbectomy-induced models, social defeat stress, hypoxia stress, chronic unpredictable mild stress, streptozotocin-induced diabetes, and tail suspension test-induced models); (2) Intervention (I): naringenin administered as the sole therapeutic agent; (3) Comparison (C): blank control groups receiving no intervention; (4) Outcomes (O): behavioral and biochemical parameters, including FST, TST, OFT, and sucrose preference test (SPT), as well as levels of DA, 5-HT, NE, 5-hydroxyindoleacetic acid (5-HIAA), IL-1β, TNF-α, BDNF, corticosterone (CORT), GSH, SOD, CAT, MDA, and NIT; and (5) Study design (S): preclinical animal studies.

The following types of studies were excluded: (1) abstracts, conference posters, reviews, meta-analyses, and case reports; (2) studies lacking a distinct naringenin-only intervention group (Excluded: studies on combined therapy or nanoformulated naringenin); (3) studies with incomplete or non-extractable data; and (4) studies not conducted in animal models.

### 2.3 Data extraction

Two independent reviewers screened the titles and abstracts to identify studies potentially relevant to the research question. After full-text selection, the included articles were systematically evaluated, and the following information was extracted using a predefined form: study identifiers (first author, year of publication, and country); animal characteristics (species, sex, and sample size); intervention details (route of administration, dosage, and treatment duration); and predefined outcome measures, including FST, TST, OFT, SPT, DA, 5-HT, NE, IL-1β, TNF-α, BDNF, CORT, GSH, SOD, CAT, MDA, 5-HIAA, and NIT. All outcome measures were analyzed using endpoint values. Data were extracted from tables and text. For unreported data, quantitative values were acquired from graphs using Engauge Digitizer. Upon completion of data extraction, a second reviewer independently verified the accuracy of all data extracted from figures. Any discrepancies were resolved via consensus discussion, and remaining disagreements were adjudicated by a third senior reviewer. If data for multiple intervention durations were presented, only those from the longest duration were included.

### 2.4 Quality assessment

The methodological quality of the included studies was assessed using the SYRCLE Risk of Bias tool for animal studies. Two independent reviewers evaluated ten core domains of bias: selection bias (sequence generation, baseline characteristics, and allocation concealment); performance bias (random housing, blinding of caregivers and investigators); detection bias (random outcome assessment and blinding of outcome assessors); attrition bias (incomplete outcome data); reporting bias (selective outcome reporting); and other potential sources of bias. To examine potential publication bias across studies, funnel plot analyses, Egger’s test and Begg’s test were performed. Each domain was judged independently by the reviewers and categorized as “low risk,” “high risk,” or “unclear risk” of bias.

### 2.5 Statistical analysis

Mean values and standard deviations (SDs) were extracted using Engauge Digitizer software when necessary. Statistical analyses were performed with Review Manager (RevMan) version 5.4. For continuous outcomes, standardized mean differences (SMDs) with corresponding 95% confidence intervals (CIs) were calculated. A p value <0.05 was considered statistically significant. Heterogeneity among included studies was assessed using the I^2^ statistic. The cut-off values were defined as follows: low heterogeneity for I^2^<25%, moderate heterogeneity for 25% ≤ I^2^ ≤ 75%, high heterogeneity for I^2^> 75%, and extremely high heterogeneity for I^2^> 85%. A significant Q test result (P < 0.05) or I^2^> 50% indicated substantial heterogeneity across studies. Given the heterogeneity among the included studies in terms of depression modeling methods, naringenin dosage, intervention duration, and outcome measures, a random-effects model was applied. The primary outcome of this study was behavioral assessments for depression. Given the generally high heterogeneity across animal experiments, we performed subgroup analyses on these main behavioral tests to further explore the sources of heterogeneity. Cut-off values for subgroup stratification were defined based on published preclinical studies. The grouping criteria are presented as follows: dose subgroups (20 mg/kg, 50 mg/kg and 100 mg/kg); treatment duration subgroups (short-term intervention: ≤2 weeks; medium-to-long-term intervention: >2 weeks); animal species subgroups (rats and mice); administration route subgroups (oral administration, intragastric gavage and intraperitoneal injection). We compared the SMD and 95%CI as well as inter-subgroup heterogeneity across all subgroups. An inter-group P < 0.05 indicated statistically significant differences in the antidepressant efficacy of naringenin among subgroups. An inter-group P ≥ 0.05 suggested that the grouping factors exerted no notable influence on the overall pooled effect. Preclinical studies are frequently affected by publication bias and methodological heterogeneity. Accordingly, we performed funnel plot analysis, Begg’s test and Egger’s test. Asymmetrical distribution of the funnel plot combined with statistically significant results of the two tests indicates publication bias arising from small-study effects or selective reporting.

## 3 Results

### 3.1 Study inclusion

**FIGURE 1:**
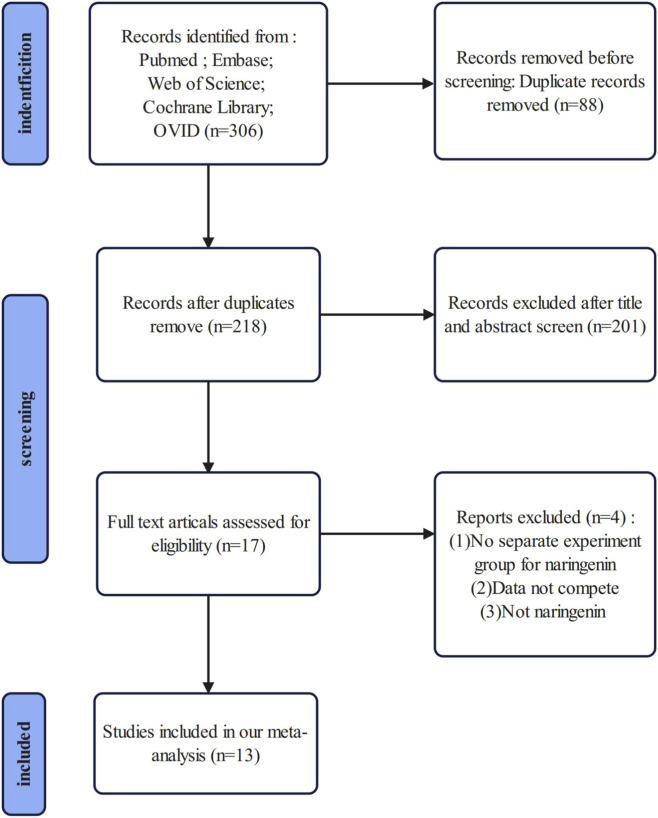
Flow chart of study searches and selection.

This study adhered strictly to the Preferred Reporting Items for Systematic Reviews and Meta-Analyses (PRISMA) guidelines for study selection. The literature screening process is illustrated in Figure 1. A comprehensive search of five major electronic databases—Embase, Web of Science, PubMed, the Cochrane Library, and OVID—yielded a total of 306 records. After automatic deduplication using EndNote, 88 duplicate entries were removed. Subsequently, 218 articles underwent independent dual screening. During title and abstract screening, 201 studies were excluded, primarily due to being non-preclinical studies, not involving depression models, or lacking naringenin intervention; inter-reviewer agreement was satisfactory. Seventeen potentially eligible articles were retrieved for full-text assessment, of which 13 studies were ultimately included in the meta-analysis (Abdelkawy et al., 2024; Bansal et al., 2018; Batool et al., 2025; El-Marasy et al., 2024; Liu et al., 2020; Olugbemide et al., 2021; She et al., 2021; Tayyab et al., 2019; Umukoro et al., 2018; Yi et al., 2010; Yi et al., 2014; Yi et al., 2012; Zhang et al., 2023). Of the four studies excluded after full-text review, one was removed because naringenin was not administered as a standalone intervention, one due to incomplete data, and two because the intervention involved naringin rather than naringenin. The included studies were published between 2010 and 2025 and were conducted by research institutions across five countries/regions. To quantitatively assess the reliability and agreement of screening results between two reviewers, Cohen’s kappa test was used to calculate the inter-rater agreement coefficient. The kappa value was 0.86 for title and abstract screening and 0.82 for full-text screening, both exceeding 0.75.

### 3.2 Study characteristics

Among the 13 included studies, three employed C57BL/6 mice, one used BALB/c mice, one involved Swiss albino mice, two used Swiss mice, three utilized Wistar rats, and three adopted Institute of Cancer Research (ICR) mice. Regarding sex distribution, 11 studies exclusively used male rodents, one study included both male and female rodents, and one study did not specify the sex of the animals. The routes of administration varied across studies: four administered naringenin via oral gavage, seven delivered it orally, and two used intraperitoneal injection. Depression models included: dexamethasone (1 study), cadmium (1 study), bilateral olfactory bulbectomy (1 study), social defeat stress (1 study), hypoxia stress (1 study), corticosterone (2 studies), chronic unpredictable stress (2 studies), streptozotocin-induced diabetes (1 study), finasteride (1 study), and tail suspension test (2 studies). Naringenin was administered at doses of 20 mg/kg, 50 mg/kg and 100 mg/kg, with treatment durations ranging from 1 day to 28 days. The formulations used comprised oral suspension (7 studies), oral solution (4 studies) and injection solution (2 studies). Detailed characteristics of the included studies are summarized in Table 7.

**TABLE 7:** Study characteristics (n = 13).

Author	Year	Country	Animal species	Sex	Sample size	Model	Drug Delivery Method	Drug duration	Outcomes
Abdelkawy	2024	Egypt	Swiss albino mice	Male	8	Dexamethasone	Oral gavage (50 mg/kg)	28 days	FST,TST,OFT,SPT,DA,5-HT,NE, IL-1β,BDNF
Batool	2025	Pakistan	Wistar rats	—	7	Cadmium	Oral gavage (100 mg/kg)	19 days	TST,TNF-α
Bansal	2018	India	BALB/c mice	Male	8	Bilateral olfactory bulbectomy (OBX)	Oral administration (100 mg/kg)	14 days	FST,SPT,5-HT,BDNF,TNF-α,CORT, GSH,SOD,CAT,MDA,NIT,5-HIAA
Umukoro	2018	Nigeria	Swiss mice	Male	6	Social defeat stress (SDS)	Intraperitioneal injection (50 mg/kg)	14 days	TST,IL-1β,TNF-α,SOD,CAT,MDA, NIT
Olugbemide	2021	Nigeria	Swiss mice	Male	7	Hypoxic stress	Intraperitioneal injection (50 mg/kg)	14 days	TST,OFT,IL-1β,BDNF,TNF-α,CORT,GSH,SOD,CAT,MDA
Liu	2020	China	C57BL/6 mice	Male	8	CORT	Oral administration (100 mg/kg)	21 days	SPT,5-HT,CORT
Zhang	2023	China	C57BL/6 mice	Male	10	CORT	Oral administration (100 mg/kg)	3 weeks	TST,SPT,DA,5-HT,NE,CORT
Tayyab	2019	India	Wistar rats	Male	6	Chronic unpre-dictable mild stress (CUMS)	Oral administration (50 mg/kg)	4 weeks	FST,OFT
El-marasy	2024	Egypt	Wistar rats	Male/female	8	Streptozotocin induced diabetic	Oral administration (50 mg/kg)	21 days	FST,DA,5-HT,NE,IL-1β,GSH,MDA 5-HIAA
She	2021	China	C57BL/6 mice	Male	12	Finasteride	Oral administration (100 mg/kg)	14 days	FST,TST,SPT,IL-1β,TNF-α,GSH, SOD,CAT,MDA,NIT
Yi	2010	China	ICR mice	Male	10	TST	Oral gavage (50 mg/kg)	1 day	FST,TST,OFT
Yi	2012	China	ICR mice	Male	8	TST	Oral gavage (20 mg/kg)	14 days	TST,OFT,DA,5-HT,NE,5-HIAA
Yi	2014	China	ICR mice	Male	8	CUMS	Oral administration (20 mg/kg)	21 days	SPT

Abbreviations:ICR, institute of cancer research; FST, forced swim test; OFT, open field test; TST, tail suspension test; SPT, sucrose preference test; 5-HT, serotonin; NE, norepinephrine; DA, dopamine; IL-1β, interleukin-1beta; BDNF, brain-derived neurotrophic factor; TNF-α, tumor necrosis factor-α; CORT, corticosterone; CAT, catalase; SOD, superoxide dismutase; GSH, glutathione; MDA, malondialdehyde; NIT, nitrite; 5-HIAA, 5-hydroxyindole acetic acid.

### 3.3 Quality assessment

The quality assessment of the 13 included studies provided detailed information regarding the methodological rigor of the selected articles. Overall, the risk of bias across studies was judged as low or unclear. Allocation concealment was unclear in all studies, indicating a potential risk of selection bias and the possibility of undetected baseline imbalances between groups. Only one study reported low risk for random housing of animals (Abdelkawy et al., 2024), and one study was assessed as low risk for investigator blinding (Yi et al., 2012), while the remaining studies were rated as unclear in these domains, suggesting a potential risk of performance bias. All studies were considered low risk with respect to incomplete outcome data. Regarding reporting bias, only one study (Abdelkawy et al., 2024)was rated as unclear for selective outcome reporting, whereas the others were judged to be at low risk, indicating a generally low likelihood of reporting bias. In terms of detection bias, four studies (El-Marasy et al., 2024; She et al., 2021; Yi et al., 2010; Yi et al., 2012)were assessed as low risk for blinding of outcome assessment, while the remainder were rated as unclear, which may have influenced the accuracy of the reported findings. These methodological considerations should be carefully taken into account when interpreting the results of the present review. However, unclear risks regarding randomization, allocation concealment and blinding may lead to overestimation of treatment effects and raise concerns about the internal validity of preclinical studies. These factors should be carefully considered when interpreting the findings of this review. The detailed information on the quality assessment of the literature is presented in Table 8.

**TABLE 8:** Quality assessment of included studies.

References	(1)	(2)	(3)	(4)	(5)	(6)	(7)	(8)	(9)	(10)
Abdelkawy et al. (2024)	Unclear	Unclear	Unclear	Low risk	Unclear	Low risk	Unclear	Low risk	Unclear	Unclear
Batool et al. (2025)	Unclear	Low risk	Unclear	Unclear	Unclear	Low risk	Unclear	Low risk	Low risk	Low risk
Bansal et al. (2018)	Unclear	Low risk	Unclear	Unclear	Unclear	Unclear	Unclear	Low risk	Low risk	Unclear
Umukoro et al. (2018)	Unclear	Low risk	Unclear	Unclear	Unclear	Unclear	Unclear	Low risk	Low risk	Low risk
Olugbemide et al. (2021)	Unclear	Low risk	Unclear	Unclear	Unclear	Low risk	Unclear	Low risk	Low risk	Low risk
Liu et al. (2020)	Low risk	Unclear	Unclear	Unclear	Unclear	Unclear	Unclear	Low risk	Low risk	Unclear
Zhang et al. (2023)	Low risk	Unclear	Unclear	Unclear	Unclear	Low risk	Unclear	Low risk	Low risk	Low risk
Tayyab et al. (2019)	Low risk	Low risk	Unclear	Unclear	Unclear	Low risk	Unclear	Low risk	Low risk	Unclear
El-Marasy et al. (2024)	Unclear	Low risk	Unclear	Unclear	Unclear	Unclear	Low risk	Low risk	Low risk	Unclear
She et al. (2021)	Unclear	Low risk	Unclear	Unclear	Unclear	Low risk	Low risk	Low risk	Low risk	Unclear
Yi et al. (2010)	Unclear	Low risk	Unclear	Unclear	Unclear	Low risk	Low risk	Low risk	Low risk	Low risk
Yi et al. (2012)	Unclear	Low risk	Unclear	Unclear	Low risk	Low risk	Low risk	Low risk	Low risk	Low risk
Yi et al. (2014)	Low risk	Unclear	Unclear	Unclear	Unclear	Unclear	Unclear	Low risk	Low risk	Unclear

(1)Random sequences generation; (2)Baseline characteristics; (3)Allocation concealment; (4)Random housing; (5)Performance blinding; (6)Random outcome assessment; (7)Blinding (for outcome evaluators); (8)Incomplete outcomes; (9)Selecting report; (10)Bias from other resources.

### 3.4 Behavioral tests

**FIGURE 2:**
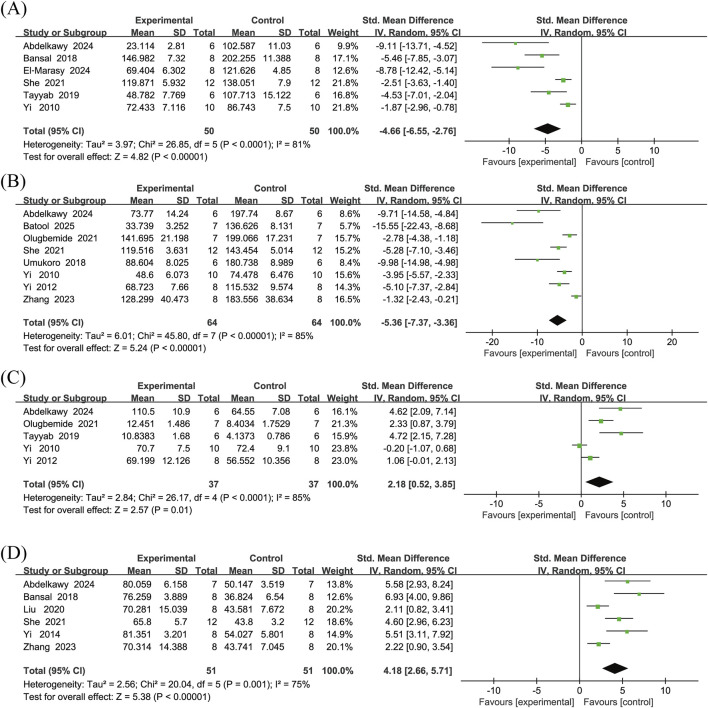
Forest plot for the effect of naringenin on the behavioral tests. **(A)** FST. **(B)** TST. **(C)** OFT. **(D)** SPT.

Meta-analysis of six studies demonstrated that naringenin significantly reduced immobility time in the FST compared with the control group [SMD = −4.66; 95%CI(-6.55, −2.76); P < 0.00001; I^2^ = 81%]. Meta-analysis of eight studies further showed that naringenin markedly decreased immobility time in the TST relative to controls [SMD = −5.36; 95%CI(-7.37, −3.36); P < 0.0001; I^2^ = 85%]. In addition, pooled analysis of five studies indicated that naringenin significantly increased the number of crossings in the OFT compared with the control group [SMD = 2.18; 95%CI(0.52, 3.85); P = 0.01; I^2^ = 85%]. Moreover, analysis of six studies revealed that naringenin significantly enhanced sucrose consumption compared with controls [SMD = 4.18; 95%CI(2.66, 5.71); P < 0.00001; I^2^ = 75%]. The forest plot showing the effect of naringenin on behavioral tests is presented in Figure 2.

### 3.5 Inflammatory markers

**FIGURE 3:**
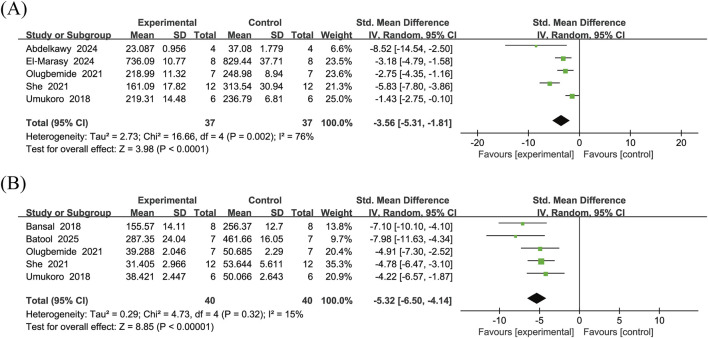
Forest plot for the effect of naringenin on the inflammatory markers. **(A) **IL-1β. **(B) **TNF-α.

Meta-analysis of five studies demonstrated that naringenin reduced IL-1β levels relative to controls [SMD = −3.56; 95%CI(-5.31,-1.81); P < 0.00001; I^2^ = 76%]. Similarly, pooled analysis of five studies showed that naringenin decreased TNF-α levels compared with the control group [SMD = −5.32; 95%CI(-6.50,-4.14); P < 0.00001; I^2^ = 15%]. The original data showed that compared with the control group, the standardized mean difference (SMD) of TNF-α in the naringenin group was −5.32 (P < 0.05), and the SMD of IL-1β was −3.56 (P < 0.05). The differences were statistically significant, indicating that naringenin could reduce the levels of IL-1β and TNF-α and participate in the regulation of inflammation. The forest plot showing the effect of naringenin on inflammatory markers is presented in Figure 3.

### 3.6 Neurotransmitter levels

**FIGURE 4:**
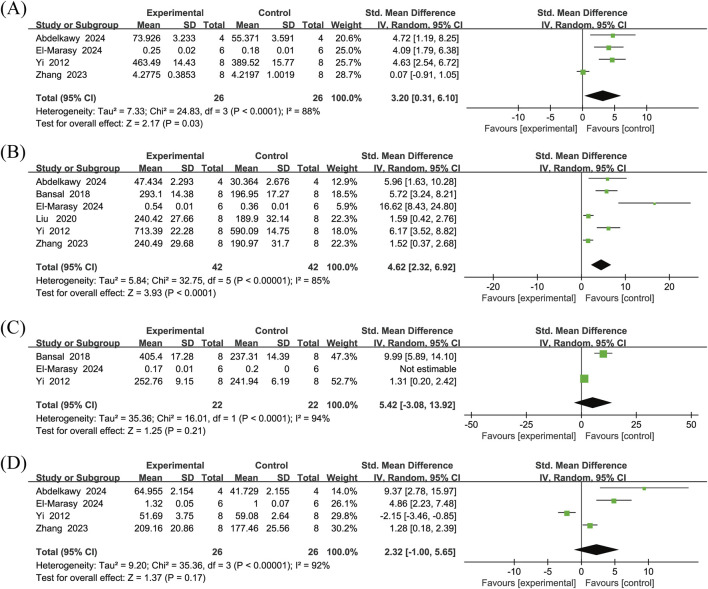
Forest plot for the effect of naringenin on the neurotransmitter levels. **(A) **NE. **(B) **5-HT. **(C) **5-HIAA. **(D) **DA.

Meta-analysis of four studies demonstrated that naringenin increased NE levels compared with the control group [SMD = 3.20; 95%CI(0.31.6.10); P = 0.03; I^2^ = 88%]. Pooled analysis of six studies further showed that naringenin elevated 5-HT levels relative to controls [SMD = 4.62; 95%CI(2.32.6.92); P < 0.0001; I^2^ = 85%]. In contrast, meta-analysis of two studies indicated no statistically significant difference between the naringenin and control groups in 5-HIAA levels [SMD = 5.42; 95%CI(-3.08.13.92); P = 0.21; I^2^ = 94%]. Similarly, pooled analysis of four studies revealed no statistically significant difference in DA levels between the two groups [SMD = 2.32; 95% CI(-1.00, 5.65); P = 0.17; I^2^ = 92%]. The forest plot showing the effect of naringenin on neurotransmitter levels is presented in Figure 4.

### 3.7 Oxidative stress markers

**FIGURE 5:**
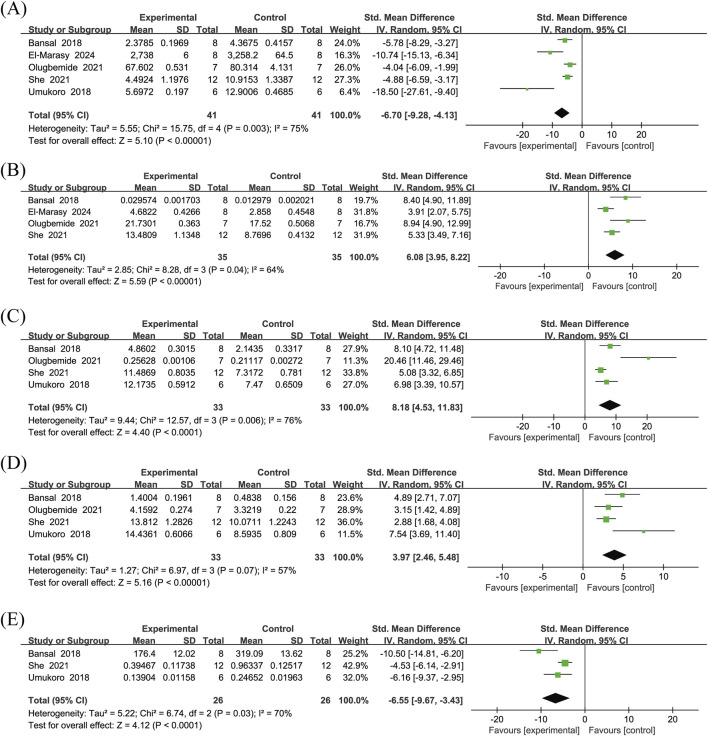
Forest plot for the effect of naringenin on the antioxident effects. **(A)** MDA. **(B)** GSH **(C)** SOD. **(D)** CAT. **(E)** nitrite.

Meta-analysis of five studies demonstrated that naringenin reduced MDA levels compared with the control group [SMD = −6.70; 95%CI(-9.28,-4.13); P < 0.00001; I^2^ = 75%]. Pooled analysis of four studies showed that naringenin increased GSH levels relative to controls [SMD = 6.08; 95%CI(3.95.8.22); P < 0.00001; I^2^ = 64%]. Similarly, meta-analysis of four studies indicated that naringenin elevated SOD levels compared with the control group [SMD = 8.18; 95%CI(4.53.11.83); P < 0.0001; I^2^ = 76%]. Analysis of four studies further demonstrated that naringenin increased CAT levels relative to controls [SMD = 3.97; 95%CI(2.46.5.48); P < 0.00001; I^2^ = 57%]. In addition, pooled results from three studies revealed that naringenin reduced NIT levels compared with the control group [SMD = −6.55; 95%CI(-9.67,-3.43); P < 0.0001; I^2^ = 70%]. The forest plot showing the effect of naringenin on oxidative stress markers is presented in Figure 5.

### 3.8 Other outcomes

**FIGURE 6:**
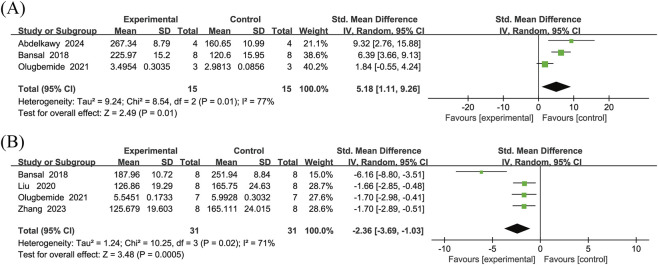
Forest plot for the effect of naringenin on the BDNF and CORT levels. **(A)** BDNF. **(B) **CORT.

Meta-analysis of three studies demonstrated that naringenin increased BDNF levels compared with the control group [SMD = 5.18; 95%CI(1.11.9.26); P = 0.01; I^2^ = 77%]. Furthermore, pooled analysis of four studies showed that naringenin reduced serum corticosterone levels relative to controls [SMD = −2.36; 95%CI(-3.69, −1.03); P = 0.0005; I^2^ = 71%]. The forest plot showing the effect of naringenin on BDNF and corticosterone levels is presented in Figure 6.

### 3.9 Subgroup analysis

Post-hoc exploratory subgroup analyses were performed stratified by dosage, intervention duration, animal species and administration route. Cut-off values for each subgroup were determined based on previous relevant literature. Interaction tests were used to compare effect differences across subgroups. Given that some subgroups included only one to four studies with limited statistical power, the subgroup results were analyzed descriptively and should be interpreted with caution. For the FST: A significant inter-subgroup difference was observed across intervention durations (P for interaction = 0.02). Interventions lasting more than 2 weeks yielded significantly better efficacy than those lasting no more than 2 weeks. No significant differences were found among subgroups stratified by dosage, animal species or administration route (all P for interaction >0.05). For the TST: There was a statistically significant difference between animal species subgroups (P for interaction = 0.003). The therapeutic effect was markedly stronger in rats than in mice. No significant inter-subgroup differences were detected for dosage, intervention duration and administration route (all P for interaction >0.05). For the OFT: Intervention duration showed a significant inter-subgroup difference (P for interaction = 0.001). Long-term intervention (>2 weeks) produced a more prominent improvement in locomotor activity. The difference across animal species subgroups reached the borderline level of statistical significance (P for interaction = 0.05), while no significant differences were found for other stratification factors. For the SPT: No significant inter-subgroup differences were observed across dosage, intervention duration and administration route (all P for interaction >0.05), indicating that the efficacy of naringenin in alleviating anhedonia was comparable under different intervention conditions. Collectively, the subgroup analyses revealed that intervention duration was the major source of heterogeneity for FST and OFT outcomes, and animal species was a key factor contributing to heterogeneity in TST results. Dosage and administration route exerted no obvious influence on the overall pooled effect. Detailed results of the subgroup analyses are shown in Table 9.

**TABLE 9:** Summary of subgroup analyses for behavioral tests.

Outcome	Stratification factor	Subgroup	N	SMD	95%CI	P value	P for interaction
FST	Dosage	50 mg/kg	4	−5.65	[-9.19,-2.11]	0.002	0.42
		100 mg/kg	2	−3.79	[-6.65,-0.93]	0.009	
	Drug duration	>2 weeks	3	−7.1	[-10.35,-3.86]	<0.0001	0.02
		≤2 weeks	3	−2.92	[-4.46,-1.39]	0.002	
	Animal species	Mice	4	−3.79	[-5.74,-1.84]	0.0001	0.26
		Rats	2	−6.44	[-10.58,-2.29]	0.002	
	Drug delivery method	Oral gavage	2	−5.14	[-12.2.1.92]	0.15	0.96
		Oral administration	4	−4.95	[-7.38,-2.51]	P < 0.0001	
TST	Dosage	20 mg/kg	1	−5.1	[-7.37,-2.84]	P < 0.0001	0.94
		50 mg/kg	4	−5.55	[-8.29,-2.81]	P < 0.0001	
		100 mg/kg	3	−5.92	[-10.57,-1.27]	0.01	
	Drug duration	>2 weeks	3	−8.37	[-17.00.0.25]	0.06	0.40
		≤2 weeks	5	−4.62	[-6.11,-3.13]	P < 0.0001	
	Animal species	Mice	7	−4.61	[-6.42,-2.79]	P < 0.0001	0.003
		Rats	1	−15.55	[-22.43,-8.68]	P < 0.0001	
	Drug delivery method	Oral gavage	4	−7.20	[-10.61,-3.79]	P < 0.0001	0.32
		Oral administration	2	−3.23	[-7.11.0.65]	0.1	
		Intraperitioneal injection	2	−5.98	[-12.99.1.04]	0.09	
OFT	Dosage	20 mg/kg	1	1.06	[-0.01.2.13]	0.05	0.24
		50 mg/kg	4	2.66	[0.20.5.11]	0.03	
	Drug duration	>2 weeks	2	4.66	[2.87.6.46]	P < 0.0001	0.001
		≤2 weeks	3	0.97	[-0.41.2.35]	0.17	
	Animal species	Mice	4	1.65	[0.04.3.27]	0.04	0.05
		Rats	1	4.72	[2.15.7.28]	0.0003	
	Drug delivery method	Oral gavage	3	1.45	[-0.50.3.40]	0.14	0.13
		Oral administration	1	4.72	[2.15.7.28]	0.0003	
		Intraperitioneal injection	1	2.33	[0.87.3.79]	0.002	
SPT	Dosage	20 mg/kg	1	5.51	[3.11.7.92]	P < 0.0001	0.32
		50 mg/kg	1	5.58	[2.93.8.24]	P < 0.0001	
		100 mg/kg	4	3.63	[1.87.5.39]	P < 0.0001	
	Drug duration	>2 weeks	4	3.53	[1.83.5.23]	P < 0.0001	0.18
		≤2 weeks	2	5.43	[3.24.7.62]	P < 0.0001	
	Drug delivery method	Oral gavage	1	5.58	[2.93.8.24]	P < 0.0001	0.31
		Oral administration	5	3.96	[2.32.5.60]	P < 0.0001	
		Unknown	1	−15.55	[-22.43,-8.68]	P < 0.0001	

FST, forced swim test; OFT, open field test; TST, tail suspension test; SPT, sucrose preference test; N, number of studies; SMD, standardized mean difference; 95%CI, 95% confidence interval.

### 3.10 Publication bias and sensitivity analysis

**FIGURE 7:**
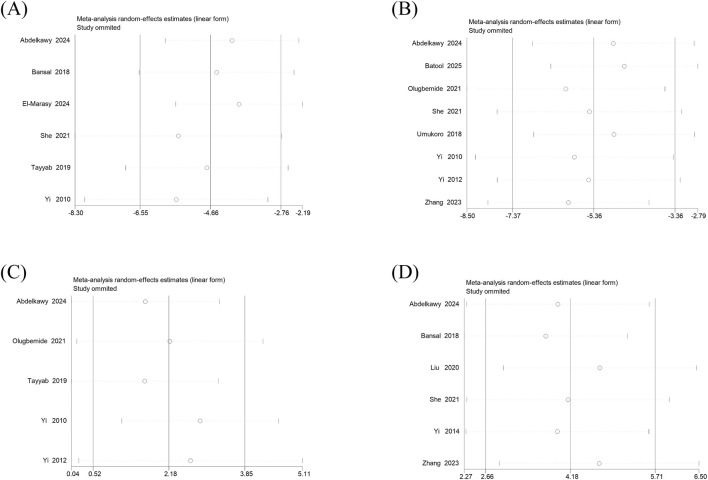
Sensitivity analysis for behavioral tests. **(A) **FST. **(B)** TST. **(C)** OFT. **(D)** SPT.

In the behavioral assessments, sensitivity analyses were performed by sequentially excluding individual studies and recalculating the pooled estimates. The overall effect sizes for the FST, TST, OFT, and SPT consistently remained within the corresponding confidence intervals, and statistical significance was preserved. These findings support the robustness of the observed antidepressant-like effects of naringenin in rodent models and indicate that the overall results were not unduly influenced by any single study, despite the presence of substantial heterogeneity. Detailed results of the sensitivity analysis for behavioral tests are presented in Figure 7. However, both Egger’s test and Begg’s test yielded P values <0.05 (see Supplementayr Supplementary Table S1), and the funnel plots exhibited asymmetry (see Supplementayr Supplementary Figure S1), suggesting the potential presence of publication bias. This publication bias may interfere with the significant pooled effect size obtained in this study and potentially overestimate the true intervention effect. This impact should be fully taken into account when interpreting the results.

## 4 Discussion

### 4.1 Summary of findings

To our knowledge, this study represents the first meta-analysis to comprehensively evaluate the effects of naringenin on depressive-like behaviors and its underlying mechanisms in rodent models. A total of 13 studies were included to systematically assess preclinical evidence regarding the antidepressant-like efficacy of naringenin. The pooled results demonstrated that naringenin significantly ameliorated depressive-like behaviors in animal models, as evidenced by reduced immobility time in the FST and TST, indicating attenuation of behavioral despair; increased crossing numbers in the OFT, reflecting enhanced exploratory activity; and elevated sucrose consumption in the SPT, suggesting improvement in anhedonia. Mechanistically, naringenin reduced the levels of pro-inflammatory cytokines, including TNF-α and IL-1β; restored monoamine neurotransmitters, such as NE and 5-HT; exerted antioxidative effects by increasing SOD, CAT, and GSH levels while decreasing MDA and NIT levels, thereby attenuating lipid peroxidation; elevated (BDNF expression; and reduced serum corticosterone concentrations. No significant effects on the levels of 5-HIAA and DA were observed. Some behavioral outcomes in this study showed abnormally high pooled effects, which may be attributed to exaggerated effect size bias caused by small-study effects, methodological biases in original studies, and publication bias. In addition, the pooled analysis of neurotransmitter indicators included a small number of studies with extremely high heterogeneity, leading to limited stability of the pooled results. Accordingly, the effects of naringenin on restoring the levels of NE and 5-HT are only preliminary estimates.

The behavioral improvements observed in this analysis are consistent with findings from previous individual animal studies. Ben-Azu et al. demonstrated that naringenin reduced immobility time in the FST and TST in mice (Ben-Azu et al., 2018). Zhang et al. reported that naringenin reversed corticosterone-induced depressive-like behaviors in mice, including decreased sucrose intake and prolonged immobility time in the TST (Zhang et al., 2023). Tayyab et al. found that naringenin alleviated reduced locomotor activity and increased despair-like behaviors in rats exposed to chronic mild stress (Tayyab et al., 2019). Accumulating preclinical studies indicate that the antidepressant effects of naringenin in rodents are mediated via multiple mechanisms. It may relieve neuroinflammation by regulating the NF-κB/BDNF pathway and downregulating MAPK14 expression (Batool et al., 2025; Olugbemide et al., 2021). Additionally, it promotes hippocampal neurogenesis through modulation of the BDNF/TrkB/CREB signaling cascade (Gao et al., 2022). Further reported that naringenin may improve depressive-like behaviors by normalizing the kynurenine/tryptophan (KYN/TRP) ratio, which was positively correlated with immobility time and negatively correlated with sucrose preference. In addition (El-Marasy et al., 2024),demonstrated that the antidepressant-like effects of naringenin were associated with increased levels of monoamine neurotransmitters, including NE, 5-HT, and DA, in the cerebral cortex and hippocampus. Other studies suggest that these behavioral improvements may also be attributed to suppression of oxidative and nitric oxide stress and reduced release of pro-inflammatory cytokines (She et al., 2021; Umukoro et al., 2018).

In terms of the anti-inflammatory mechanism, this study revealed that naringenin reduced the levels of pro-inflammatory cytokines, including TNF-α and IL-1β, which may be associated with downregulation of the NF-κB signaling pathway (Olugbemide et al., 2021; Sarubbo et al., 2018). Park et al. found that naringenin inhibits the activation of both NF-κB and MAPK pathways, thereby reducing the release of lipopolysaccharide induced inflammatory cytokines. (Park et al., 2012). Studies have shown that naringenin inhibits the activation of the NLRP3 inflammasome and blocks the downstream inflammatory cascade. (Chen et al., 2019; El-Marasy et al., 2024). Studies have demonstrated that naringenin may alleviate neuroinflammation, protect neurons, and improve synaptic plasticity and nerve conduction via the NLRP3/NF-κB pathway, thereby ameliorating depressive- and anxiety-like behaviors in rodents (Wang et al., 2020; Zhu and Gao, 2024). These findings are consistent with the results of the present study.

The kynurenine pathway is recognized as a critical link between neuroinflammation and depression. Inflammatory stress shifts tryptophan metabolism toward the kynurenine branch, depleting the precursor of serotonin and further exacerbating the disturbance of monoamine neurotransmitters (Correia and Vale, 2022; Dowlati et al., 2010; Shi et al., 2025). Several studies have demonstrated that naringenin increases neurotransmitter levels in rat brain tissue, thereby exerting neuroprotective effects against brain injury (Bahramsoltani et al., 2015; Mansour et al., 2023; Sarubbo et al., 2018). These findings are consistent with our results, as the present meta-analysis showed that naringenin restored NE and 5-HT levels in the brains of rodents (Abdelkawy et al., 2024; Yi et al., 2012; Zhang et al., 2023). From the perspective of molecular regulation, naringenin markedly inhibits MAO-A activity (Haidary et al., 2026)), upregulates the expression of tryptophan hydroxylase (Sarubbo et al., 2018), thereby preserving monoaminergic neurotransmitter levels and attenuating oxidative stress (Sachdeva et al., 2015).

Oxidative stress imbalance is an important pathological mechanism of depression. Naringenin exerts antioxidant protective effects in rodents, as evidenced by increased levels of SOD, CAT, and GSH(Zhang et al., 2022; Zhu and Gao, 2024), along with reduced concentrations of MDA and NIT, indicating attenuation of lipid peroxidation (Sadeghi Nejad et al., 2023; She et al., 2021). Naringenin restores oxidative stress–related parameters and preserves the integrity of the antioxidant defense system (Rai, Jat, et al., 2024; Rai, Kalar, et al., 2024). These findings are consistent with the present study. The antioxidant properties of naringenin are largely attributable to its hydroxyl substituents, which exhibit high reactivity toward reactive nitrogen species and ROS (Cavia-Saiz et al., 2010). Furthermore, naringenin mitigates inflammation and apoptosis by modulating NF-κB signaling in neural tissues (Mansour et al., 2023). Through these mechanisms, Reported that the antioxidant effects of naringenin may be associated with normalization of the KYN/TRP ratio. The KYN/TRP ratio was positively correlated with oxidative stress markers, such as NIT, and negatively correlated with endogenous antioxidants, including GSH, SOD, and CAT (Bansal et al., 2018).

Downregulation of BDNF and hyperactivity of the HPA axis are two key pathological characteristics of depression. The present study demonstrated that naringenin significantly increased BDNF levels in the brains of rodents, thereby promoting the repair of damaged neurons. This effect may be associated with the activation of the BDNF/TrkB/CREB pathway and the upregulation of BDNF expression in the hippocampus. (Ahmad et al., 2021; Olugbemide et al., 2021; Tayyab et al., 2019; Yi et al., 2014; Zhang et al., 2023; Zhu and Gao, 2024). These findings are consistent with the majority of previous studies. Meanwhile, naringenin reduces serum corticosterone levels and suppresses excessive activation of the HPA axis. This effect may be related to its capacity to decrease proinflammatory cytokines, including IL-6, TNF-α, and interferon-γ, in rodents (Bansal et al., 2018), as well as to modulate tryptophan metabolism, thereby improving 5-HT secretion (Liu et al., 2020).

However, the included studies in this analysis presented high heterogeneity and were mostly exploratory. All regulatory pathways proposed so far remain hypothetical, and no consistent and definitive conclusions have been reached. Therefore, it cannot be concluded that naringenin exerts definite antidepressant effects through a single pathway.

Flavonoids are ubiquitous polyphenolic secondary metabolites in plants, mainly exerting antioxidant, anti-inflammatory, neuroprotective, cardioprotective and metabolic regulatory effects. Common members including quercetin, curcumin, resveratrol and luteolin can modulate neurotransmitter levels, promote neuronal regeneration, ameliorate HPA axis dysfunction, relieve inflammation and mitigate oxidative stress. Their functional targets largely overlap with those of naringenin (Moore et al., 2018; Zhang et al., 2020; Zhou et al., 2025). Nevertheless, their major limitations include the scarcity of clinical trials, low oral bioavailability, and a lack of direct evidence supporting clinical application.

### 4.2 Strengths and limitations

#### 4.2.1 Strengths of the study

To our knowledge, this study is the first to systematically evaluate the preclinical evidence regarding the antidepressant effects of naringenin through a systematic review and meta-analysis. The study was conducted in accordance with the PICOS framework and employed a comprehensive search strategy to minimize the risk of omitting relevant studies. By quantitatively synthesizing fragmented and small-sample animal data, the meta-analysis generated statistically robust effect estimates. Beyond assessing depressive-like behaviors, we systematically examined potential underlying mechanisms, including inflammation, neurotransmitter regulation, oxidative stress, BDNF expression, and corticosterone levels. This integrative approach extends beyond behavioral phenotypes to elucidate molecular and biochemical pathways, thereby providing multidimensional mechanistic support for the antidepressant potential of naringenin and highlighting its translational relevance.

#### 4.2.2 Limitations of the study

##### 4.2.2.1 The included studies have limitations in overall quality and sample size

A total of 13 preclinical animal studies involving 212 experimental animals were enrolled in this analysis. The overall sample size was small, leading to notable small-study effects and limited external validity. In addition, all included studies were animal experiments with no supporting data from human clinical trials.

##### 4.2.2.2 Animal behavioral models have considerable limitations in clinical translation

The FST, TST, OFT and SPT are widely used behavioral paradigms in preclinical antidepressant research. However, animal depression models cannot fully recapitulate the complex and heterogeneous pathological features of human depression. Accordingly, behavioral outcomes in animals cannot be directly equated with clinical depressive symptoms in humans, creating inherent barriers to the clinical translation of experimental findings.

##### 4.2.2.3 The sources of high heterogeneity across studies have not been fully resolved

Substantial high heterogeneity was observed in multiple pooled analyses, with most I^2^ values exceeding 75%. Although subgroup analyses were performed, the heterogeneity could not be fully explained. Potential confounding factors include differences in animal species and strains, diverse depression modeling protocols, as well as inconsistent dosage, administration routes and intervention durations of naringenin.

##### 4.2.2.4 There was a marked gender bias in the included studies

The vast majority of experiments only used male rodents. Given that the incidence of depression is significantly higher in females than in males, and sex hormones directly regulate central inflammation, the HPA axis and neurotransmitter pathways, data obtained solely from male animals greatly reduce the generalizability of the conclusions.

##### 4.2.2.5 Naringenin exhibits inherent pharmacokinetic deficiencies

It has poor water solubility, undergoes extensive intestinal first-pass metabolism, and is characterized by low oral absorption, low blood-brain barrier permeability and rapid *in vivo* clearance, resulting in extremely low oral bioavailability, which greatly restricts its clinical application. Although preliminary explorations have been conducted on formulation optimization strategies such as nanocarriers, liposomal delivery systems and prodrugs, no mature and practical delivery systems are currently available.

##### 4.2.2.6 Potential publication bias was detected

The results of Egger’s test, Begg’s test combined with funnel plots indicated the presence of potential publication bias in this study. Positive efficacy outcomes tend to be published preferentially, which may ultimately overestimate the improvement effect of naringenin on depressive behaviors. Future studies should incorporate grey literature and unpublished data as much as possible to mitigate the interference caused by publication bias.

## 5 Conclusion

Available preclinical studies suggest that naringenin alleviate depressive-like behaviors in rodents, potentially through multiple mechanisms, including inflammation suppression, restoration of monoamine neurotransmitter homeostasis, mitigation of oxidative stress injury, upregulation of BDNF expression and modulation of the overactive HPA axis. Nevertheless, due to high methodological heterogeneity, small sample sizes and potential publication bias among the included studies, these multi-target regulatory mechanisms remain tentative hypotheses without definitive verification. Poor *in vivo* bioavailability of naringenin hinders its clinical translation. Although various delivery formulations have been preliminarily investigated, no mature solutions are available so far. Additionally, all current evidence is derived from animal experiments. High-quality human clinical trials are urgently needed to clarify its pharmacokinetic profiles and therapeutic efficacy.

In conclusion, naringenin is a promising plant-derived antidepressant compound. Further standardized basic experiments, formulation optimization studies and rigorous clinical trials in humans are still required to evaluate its safety, efficacy and optimal dosage in patients with depression, and to provide references for the research and development of natural flavonoid antidepressants.

## Data Availability

The original contributions presented in the study are included in the article/Supplementary Material, further inquiries can be directed to the corresponding author.

## Supplementary material

SUPPLEMENTARY FIGURE S1 Funnel plots for behavioral tests. **(A)** FST; **(B)** TST; **(C)** OFT; **(D)** SPT.

SUPPLEMENTARY TABLE S1 Publication bias for behavioral tests.
