# Supplementary figures and images for: Antidepressant effects and therapeutic potential of naringenin: a systematic review and meta-analysis of preclinical studies
Source: Front Pharmacol. 2026 Jun 24;17:1836030. doi: 10.3389/fphar.2026.1836030 (PMC13342214; doi:10.3389/fphar.2026.1836030)

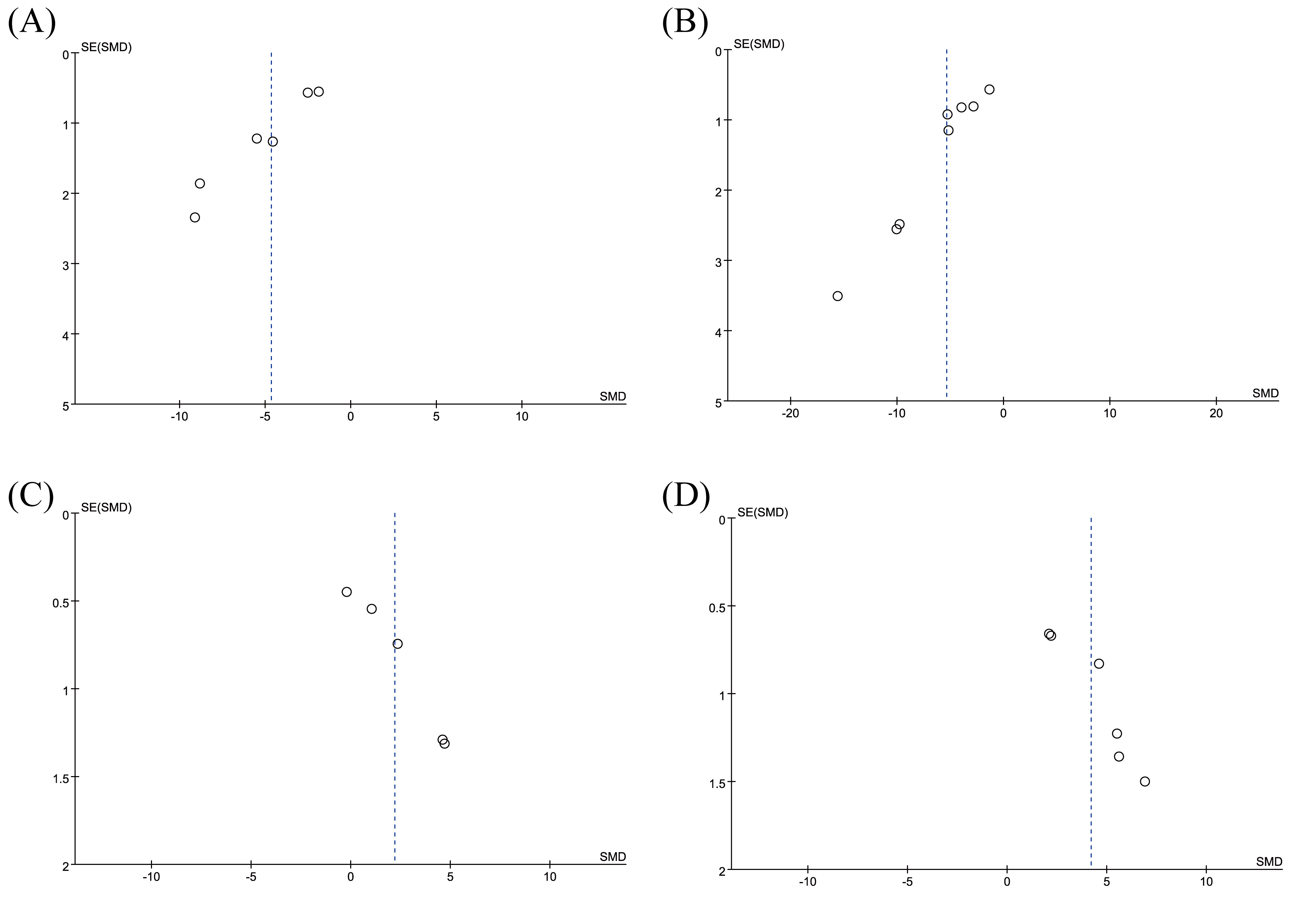

Supplement: Supplementary file 1 [file Image1.tif]
