# Supplementary material for: Antidepressant effects and therapeutic potential of naringenin: a systematic review and meta-analysis of preclinical studies
Source: Front Pharmacol. 2026 Jun 24;17:1836030. doi: 10.3389/fphar.2026.1836030 (PMC13342214; doi:10.3389/fphar.2026.1836030)
Supplement: Supplementary file 2 [file Table1.doc]

Table S1. Publication bias for behavioral tests

| outcomes | Egger's test | | | Begg's Test | |
| --- | --- | --- | --- | --- | --- |
|  | n | t | p | z | p |
| FST | 6 | -8.63 | 0.001 | -2.07 | 0.039 |
| TST | 8 | -6.3 | 0.001 | -3.22 | 0.001 |
| OFT | 5 | 6.82 | 0.006 | 2.45 | 0.014 |
| SPT | 6 | 4.97 | 0.008 | 2.44 | 0.015 |

Notes:N means number of studies
